# Microbubbles combined with ultrasound therapy in ischemic stroke: A systematic review of in-vivo preclinical studies

**DOI:** 10.1371/journal.pone.0191788

**Published:** 2018-02-08

**Authors:** Laurent Auboire, Charles A. Sennoga, Jean-Marc Hyvelin, Fréderic Ossant, Jean-Michel Escoffre, François Tranquart, Ayache Bouakaz

**Affiliations:** 1 UMR Imagerie et Cerveau, Inserm U930, Université François-Rabelais de Tours, France; 2 CHRU de Tours, Service d’échographie-Doppler, Tours, France; 3 Bracco Suisse SA, Geneva, Switzerland; 4 CHRU de Tours, CIC-IT, Tours, France; 5 Advice-US Consulting, Nernier, France; Cedars-Sinai Medical Center, UNITED STATES

## Abstract

**Background:**

Microbubbles (MBs) combined with ultrasound sonothrombolysis (STL) appears to be an alternative therapeutic strategy for acute ischemic stroke (IS), but clinical results remain controversial.

**Objective:**

The aim of this systematic review is to identify the parameters tested; to assess evidence on the safety and efficacy on preclinical data on STL; and to assess the validity and publication bias.

**Methods:**

Pubmed^®^ and Web of Science^TM^ databases were systematically searched from January 1995 to April 2017 in French and English. We included studies evaluating STL on animal stroke model. This systematic review was conducted in accordance with the PRISMA guidelines. Data were extracted following a pre-defined schedule by two of the authors. The CAMARADES criteria were used for quality assessment. A narrative synthesis was conducted.

**Results:**

Sixteen studies met the inclusion criteria. The result showed that ultrasound parameters and types of MBs were heterogeneous among studies. Numerous positive outcomes on efficacy were found, but only four studies demonstrated superiority of STL versus recombinant tissue-type plasminogen activator on clinical criteria. Data available on safety are limited.

**Limitations:**

Quality assessment of the studies reviewed revealed a number of biases.

**Conclusion:**

Further in vivo studies are needed to demonstrate a better efficacy and safety of STL compared to currently approved therapeutic options.

**Systematic review registration:**

http://syrf.org.uk/protocols/

## Introduction

Ischemic stroke (IS), described as death of neuronal cells due to arrest of regional cerebral blood flow by intra-arterial thrombi, is the second most common cause of death and the first leading cause of disability worldwide [1]. Although intravenous (IV) administration of recombinant tissue-type plasminogen activator (rtPA), the only thrombolytic agent approved for the reperfusion of occluded arteries, shows improved outcomes in terms of mortality and disability in IS patients, its use is characterized by increased incidences of hemorrhage [2]. Patients ineligible for rtPA or failing to recanalize with rtPA are now treatable using thrombectomy. However, thrombectomy is presently used only at comprehensive stroke centers and limited to an artery with a diameter > 2 mm [3, 4]. Use of other thrombolytic drugs (e.g., tenecteplase) were previously trialled, but showed no beneficial improvements in treatment outcomes when compared with rtPA [5].

Sonothrombolysis (STL), involving the use of microbubbles (MBs) combined with transcranial ultrasound (US) to potentiate clot dissolution with or without administration of rtPA, is under clinical evaluation as a method for the recanalization of occluded cerebral arteries [6–8]. A meta-analysis of clinical STL shows no beneficial treatment outcome in terms of mortality and disability at 3 months versus approved treatment (rtPA). Patients also experience a higher rate of cerebral hemorrhage [9]. Another meta-analysis of randomized controlled trials and case-control studies concluded that sonothrombolysis with microbubbles is a safe and effective treatment in ischemic stroke [10]. A more recent clinical trial, NOR-SASS (Norwegian Sonothrombolysis in Acute Stroke Study), which was prematurely interrupted (lack of funding), concluded that the use of STL is safe as a thrombolytic therapy and with no significant adverse effects [8].

Twenty years after the demonstration by Tachibana et al. that STL with microbubbles has a thrombolytic effect, numerous studies have been performed in Vivo [11]. If comprehensive review on the subject are available [12–15], no systematic review on in-vivo studies has been performed yet. Such review is required to summarize the evidence accumulated, to provide transparency on the quality of these studies and help to design new studies, which can ease clinical translation. This systematic review will focus on the association of microbubbles and ultrasound for sonothrombolysis. Other approaches such as histotripsy or sonothrombolysis without microbubbles will not be included in this review to maintain a homogeneity in the parameters tested.

The purpose of this systematic review is (i) to identify the parameters tested and their results, (ii) to assess evidence on the safety and efficacy from preclinical data on STL, and (iii) to assess the validity of the reviewed study and identify publication bias.

## Methods

Pubmed^®^ and Web of Science^TM^ electronic databases were screened by two of the authors (LA, CAS) using pre-defined search terms (January 1995–April 2017) for preclinical in vivo reports employing STL or STL combined with rtPA (rtPA-STL) in IS. The search terms ((stroke [MeSH Terms] OR fibrinolytic drugs [MeSh Terms] OR thrombolytic OR thrombolytic agents [MeSH Terms] OR thrombolytic drugs [MeSH Terms]) AND (microbubbles [MeSH Terms] OR microspheres [MeSH Terms]) AND ("French" [language] OR "English" [language])) OR “sonothrombolysis” were used on the Pubmed^®^ database. The search terms (“thrombolytic therapy OR stroke”) AND (“microbubbles OR microspheres”) OR “sonothrombolysis”, (“ultrasound contrast agents AND stroke”) were used on the Web of Science^TM^ database. The search term “ultrasound AND thrombolysis” was used on both databases. The full electronic search strategy for the two databases are available in S1 File. The inclusion and exclusion criteria are summarized in Table 1. Data were extracted by two of the authors (LA, CAS) following a pre-defined schedule including: species, stroke model, type of clot, duration of clotting, duration of ischemia, ultrasound parameters (frequency, duty cycle, duration of insonation, position of the ultrasound transducer, acoustic power, and peak negative pressure), microbubbles characteristics (type, route of administration, thrombolytic drug associated, volume of injection, and concentration of microbubbles per kilogram), significant outcomes, and quality evaluation. The Collaborative Approach to Meta-Analysis and Review of Animal Data from Experimental Studies (CAMARADES) was used to assess the quality and risk of bias of the studies included [16]. This systematic review was written in accordance with the PRISMA guidelines [17]. The protocol was registered on the CAMARADES-NC3Rs Preclinical Systematic Review & Meta-analysis Facility (SyRF) and is available online (https://drive.google.com/file/d/0B7Z0eAxKc8ApWi1HY3lvNU1pU2M/view).

**Table 1:** Inclusion and exclusion criteria used to select studies.

Inclusion Criteria	Exclusion Criteria
Concerning Stroke	Sonothrombolysis without microbubbles
Involving microbubbles	Review papers
On animals	Comments, Letters
French or English	Studies concerning other arteries not directly supplying the brain (List of arteries included: cerebral artery, carotid artery, vertebral artery, *rete mirabele* and pharyngeal artery in pig)
With Thrombolytic drugs or not	Neuro-protective study without thrombolysis
	Studies involving humans
	*In vitro* studies

## Results

### Studies selected

**Fig 1:**
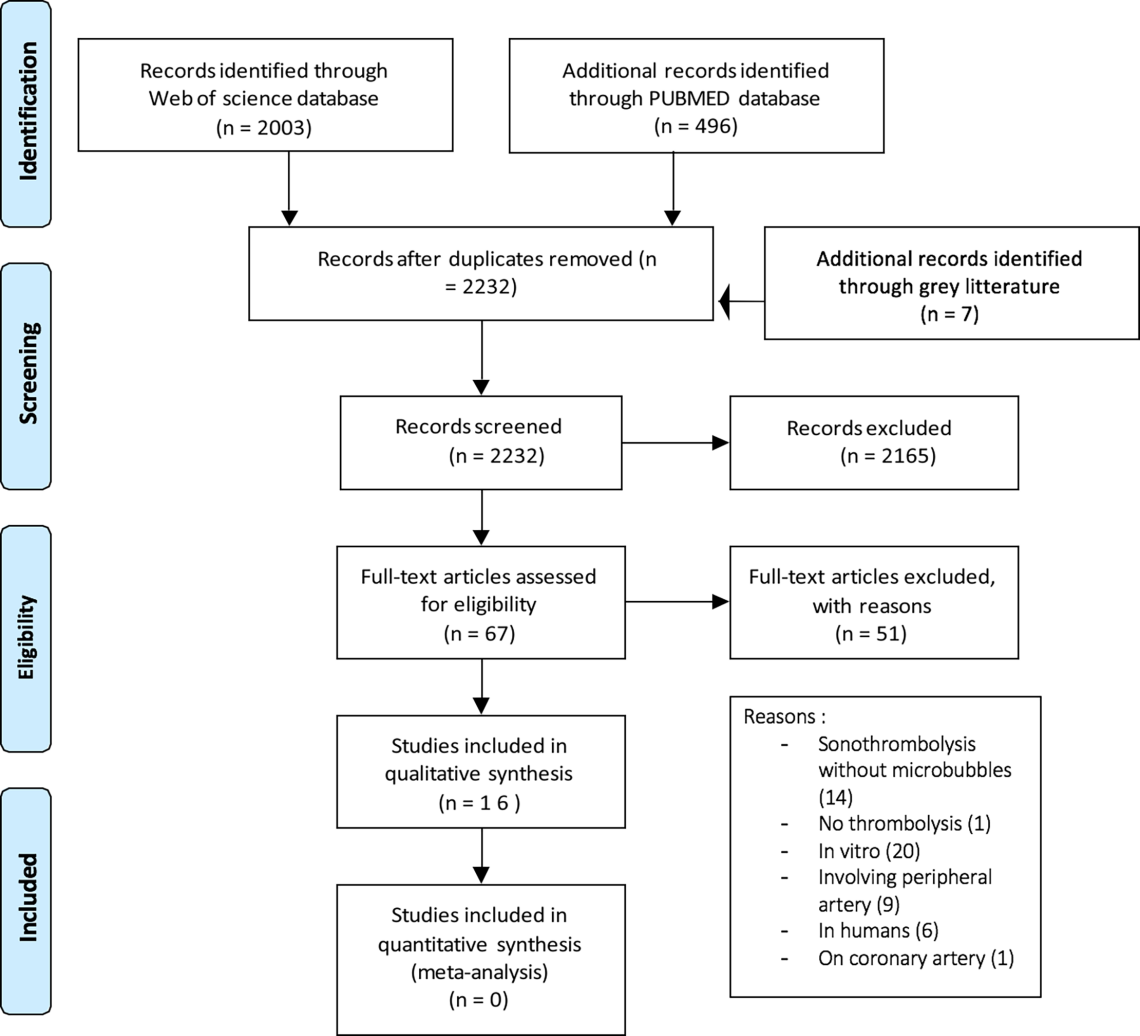
PRISMA 2009 flow diagram. Extracting schedule of the studies.

Sixteen studies met the inclusion criteria (Table 2). The selection schedule used is illustrated in Fig 1.

**Table 2:** General description of studies that fulfilled the inclusion/exclusion criteria.

**5 studies focused only on STL treatment efficacy**	1. Moumouh et al. (2009), with microbubbles (SonoVue^®^) alone 2. Culp et al. (2003), with lipid shelled microbubbles alone 3. Wang et al. (2008), with targeted microbubbles (eptifibatide) alone 4. Alonso et al. (2009), with targeted microbubbles (abciximab) alone 5. Culp et al. (2004), with targeted microbubbles (eptifibatide) alone
**4 studies focused on safety and efficacy of STL**	1. Culp et al. (2011), with targeted or non-targeted microbubbles (Definity^®^, or custom 3 μm microbubbles), or targeted microbubbles (eptifibatide) alone 2. Liu et al. (2012), with microbubbles (SonoVue^®^) with Urokinase 3. Ren et al. (2012), with targeted microbubbles (eptifibafide) alone 4. Gao et al. (2014) with microbubbles (Definity^®^) alone, with the objective of determining the type and level of cavitation required to dissolve thrombi and improve cerebral blood flow
**2 studies focused on rtPA-STL treatment efficacy**	1. Tomkins et al. (2015) with lipid shelled microbubbles (BR-38^®^) and rtPA on a new *in-vivo* model using platelet rich plasma clot 2. Ren et al. (2015), tested the efficacy of rtPA-STL for thrombolysis.
**5 Studies focused on efficacy and safety of rtPA-STL**	1. Brown et al. (2011), with Definity^®^ or albumin/dextrose microbubbles and low dose of rtPA, addressed the possibility of reducing the dose of rtPA and decreasing the rate of intracerebral hemorrhages 2. Flores et al. (2013), with microbubbles (Definity^®^) alone and microbubbles with rtPA 3. Lu et al. (2016), attempted to determine whether STL or rtPA-STL could dissolve platelet rich or erythrocyte rich microthrombi. 4. Schleicher et al. (2016) and Nedelmann et al. (2010) tested the ability of STL or rtPA-STL to improve microvascular patency after a transient vascular occlusion.

### Animal models and clots

Thrombi (0.1 mL–1 mL) were delivered into the ascending pharyngeal artery and rete mirabile using femoral catheterization in a swine model [18–20] and arterial occlusions assessed using angiography [18, 19] or MRI [20]. Gao et al. [20] achieved bilateral arterial occlusions, because the right and the left ascending pharyngeal arteries supply the rete mirabile. Acute arterial occlusions, induced by blocking the right middle cerebral artery with autologous blood clots delivered by femoral catheterization, were used in a rabbit model [21–26]. Finally, five studies used a rat model of stroke with varying modifications [27–31]. Alonso et al. [27] injected 4 mm long human blood clots through the external carotid artery (ECA) and delivered them into a pre-ligated common carotid artery (CCA). Moumouh et al. [29] injected 500 μm of autologous blood clots directly into the CCA. Tomkins et al. [31] administered 30 mm plasma-rich clots into the internal carotid artery via the ECA and assessed vascular occlusion using laser Doppler velocimetry. Ren et al. [30] introduced 0.6 mm × 0.08 cm autologous clots into the left CCA. Lu et al. [28] injected white or red micro-thrombi into a distally ligated ECA after clamping the CCA. The clamp was subsequently removed and microthrombi were liberated into the cerebral vasculature. Nedelmann et al. and Schleicher et al. [32, 33] induced a transient vascular occlusion by introducing a filament through the carotid artery in the middle cerebral artery. Table 3 summarizes the clot compositions, clotting time prior to injection, duration following in vivo vessel occlusion, and initiation of thrombolytic treatment employed.

**Table 3:** Summary of compositions of various clots, clotting time before injection, and time between injection and the start of treatment.

References	Species	Clot type	Clotting time before injection (min)	Time between injection of blood clot and the start of treatment (min)
**Culp et al. (2003)**	Swine	Autologous whole blood	217	Immediately
**Culp et al. (2004)**	Swine	Autologous whole blood	276	Immediately
**Gao et al. (2014)**	Swine	Autologous whole blood	NA	15
**Alonso et al. (2009)**	Rat	Heterologous whole blood (human)	120	Immediately
**Moumouh et al. (2010)**	Rat	Autologous whole blood	30	240
**Tomkins et al. (2015)**	Rat	Autologous platelet rich clot	Approx. 24H	60
**Wang et al. (2008)**	Rabbit	Autologous whole blood	226	30
**Culp et al. (2011)**	Rabbit	Autologous whole blood	4320	60
**Brown et al. (2011)**	Rabbit	heterologous whole blood (human)	180 à 300	Immediately
**Flores et al. (2011)**	Rabbit	Heterologous whole blood (human)	180	Immediately
**Ren et al. (2012)**	Rabbit	Autologous whole blood	120 à 360	360
**Liu et al. (2012)**	Rabbit	Autologous whole blood	30	30
**Ren et al. (2015)**	Rat	Autologous whole blood	180	NA
**Lu et al. (2016)**	Rat	Autologous platelet and erythrocyte rich thrombi	205	5
**Nedelmann et al. (2010)**	Rat	Filament occlusion–microvascular impairment	NA	100
**Schleicher et al. (2016)**	Rat	Filament occlusion–microvascular impairment	NA	100

### Sonothrombolysis

Tables 4 and 5 summarize the MB type (targeting or non-targeting to receptors expressed on thrombi including the GP2B/3A: glycoprotein 2B/3A is an integrin expressed on platelets which will bind fibrinogen during platelet activation leading to an aggregation of them as they may connect to the same fibrinogen molecule), concentration, route of administration, presence or absence of thrombolytic drugs, and US parameters (e.g., frequency, duty cycle, acoustic pressure, and exposure time) employed for the reviewed STL studies.

**Table 4:** Summary of the type of microbubbles used including their dosage, administration route, protocols of injection by type of microbubbles (MBs/TMBs), and combination with thrombolytic drugs.

	MB			TMB					MB or TMB associated with thrombolytic drugs							
**References**	Culp et al. (2003)	Moumouh et al. (2010)	Gao et al. (2014)	Culp et al. (2004)	Wang et al. (2008)	Alonso et al. (2009)	Culp et al. (2011)	Ren et al. (2012)	Nedelmann et al. (2010)	Brown et al. (2011)	Flores et al. (2011)	Liu et al. (2012)	Tomkins et al. (2015)	Ren et al. (2015)	Schleicher et al. (2016)	Lu et al. (2016)
**Animal model**	Autologous clot in a swine model	Autologous clot in a rat model	Autologous clot in a swine model	Autologous clot in a swine model	Autologous clot in a rabbit model	Human clot in a rat model	Autologous clot in a rabbit model	Autologous clot in a rabbit model	Filament occlusion in a rat microvascular impairment	Human clot in a rabbit model	Human clot in a rabbit model	Autologous clot in a rabbit model	Autologous white clot in a rat model	Autologous clot in a rat model	Filament occlusion in a rat model microvascular impairment	Autologous red and white clot in a rat model
**Microbubbles type**	Optison®	Definity®	Definity®	Decafluorobutane targeted microbubbles GP2B3A, eptifibatide	Targeted Microbubbles GP2B/3A, SF6 gas	Targeted Microbubbles GP2B/3A abciximab	Definity® or 3micronMB, 3microMB targeted on GP2B3A	Targeted Microbubbles GP2B/3A	Sonovue®	Definity®	Definity®	SonoVue®	BR38®	Sonovue®	BR38® or Sonovue®	Lipid shelled perfluoropropane microbubbles
**Route of administration**	IA	IV	IV	IV	IV or IA	IV	IV	IA (intra-arterial catheter) or IV	IV	IV	IV	IV	IV	IV	IV	IV
**Type and dose of thrombolytic drugs**	None	None	None	None	None	None	None	None	rtPA, 10 mg/kg	rtPA, 0.1 to 0.9 mg/kg	rtPA, 0.9mg/kg	Urokinase, 20000 UI/ kg	rtPA, 10 mg/kg	rtPA, dosage not precised	rtPA, 10 mg/kg	rtPA, 5 or 10mg/kg
**Type of injection**	Bolus	Bolus	Infusion	Infusion	Bolus	Infusion	Bolus	Bolus	4 Bolus	Bolus	Bolus	Bolus of MB, Infusion of urokinase	Bolus of MB, Bolus+infusion of RtPA	Infusion	4 Bolus	Infusion
**Volume**	4.5mL (1mL + 0.5 mL every 3 min)	0.6mL (0.2mL every 10 min)	90mL	15 mL	5mL	5mL (0.2mL/min)	6mL	5mL (1mL x 5)	0.4mL (0.1mLx4)	6 mL	6mL	2mL	0.4 mL (0.1mL every 15 min)	1mL	0.4mL (0.1x4)	0.24mL
**Concentration of Microbubbles (MB/kg), average weight of animals**	0.75–1.2x10^8MB/kg (30kg)	1.92x10^9MB/kg (240-280g)	Not available (33.9kg)	4.8x10^9MB/Kg (30kg)	5.5x10^8MB/kg (3kg)	4.48x10^10 MB/kg (446g)	Definity® MB: 1.92x10^9MB/kg For Albumin MB alone or tagged: 9.61x10^9^ MB/Kg (5.2kg)	3.3x10^8MB/kg	9x10^6MB/Kg (311g)	1.92x10^9MB/kg (5.2kg)	1.92x10^9MB/kg (5.2kg)	0.6–3.2x10^8MB/kg (3.1kg)	4x10^8MB/mL 13.3x10^8MB/kg (300g)	1.7–8.6x10^7MB/Kg (530-620g)	9x10^6MB/kg (350g) for Sonovue®, 1.8x10^4MB/kg for BR38® (for full dose)	Not available

**Table 5:** Summary of the US parameters utilized in the different studies.

References	Culp et al. (2003)	Culp et al. (2004)	Wang et al. (2008)	Alonso et al. (2009)	Moumouh et al. (2010)	Nedelmann et al. (2010)	Culp et al. (2011)	Brown et al. (2011)	Flores et al. (2011)	Ren et al. (2012)	Liu et al. (2012)	Gao et al. (2014)	Tomkins et al. (2015)	Ren et al. (2015)	Lu et al. (2016)	Schleicher et al. (2016)
**Animal model**	Autologous clot in a swine model	Autologous clot in a swine model	Autologous clot in a rabbit model	Human clot in a rat model	Autologous clot in a rat model	Filament occlusion in a rat model microvascular impairment	Autologous clot in a rabbit model	Human clot in a rabbit model	Human clot in a rabbit model	Autologous clot in a rabbit model	Autologous clot in a rabbit model	Autologous clot in a swine model	Autologous white clot in a rat model	Autologous clot in a rat model	Autologous red and white clot in a rat model	Filament occlusion in a rat model microvascular impairment
**Frequency**	1 MHz	1MHz	800 kHz	2MHz	2MHz	1 to 3 MHz	1MHz	1MHz	1MHz	800kHz	2MHz	1.6MHz	3MHz	1MHz	2MHz	3MHz
**Transducer geometry**	Focused (5 mm) cylindrical beam 10 cm2	Focused (5 mm) cylindrical beam 10 cm2	2 cm diameter transducer	Imaging probe (P4-2, Philips®) focused (5cm)	Doppler probe (DWL®)	Imaging probe (Sonos 7500; Philips®)	Focused (5 mm) cylindrical beam 10 cm2	Focused (5 mm) cylindrical beam 10 cm2	Focused (5 mm) cylindrical beam 10 cm2	transducer of 2.8cm2	Doppler probe (DWL®), 25-30mm depth, sampling volume of 10 mm^3^	Imaging probe (S5-1, Philips®) focused (3cm)	Imaging probe (Sonos 7500; Philips®)	Imaging probe (S5-1, Philips®)	Imaging probe (4V1C Acuson® sequoia)	Imaging probe (Sonos 7500; Philips®), focused at 57 mm.
**Position of US probe**	Temporal bone	Temporal bone	Behind the eye	On carotid	Parietal bone	NA	Temporal bone	Temporal bone	Temporal bone	On carotid	Right parietal bone	Temporal bone	40 mm above the skull	Carotid through a human temporal bone	Temporal	NA
**Duty cycle**	20%	20%	20%	5%	1%	NA	20%	20%	20%	20%	NA	0.01% or 0.04%	NA	NA	NA	NA
**Power**	2.2W/cm^2^ (Isata)	2W/cm^2^ (Isata)	2.4W/cm^2^	NA	500mW/cm^2^ (Ispta)	NA	0.8W/cm^2^ (Isata)	0.8W/cm^2^ (Isata)	0.8W/cm^2^ (Isata)	2.787W/cm^2^	252mW/cm^2^ (Ispta)	NA	NA	NA	NA	NA
**Negative peak pressure**	NA	NA	NA	1.56 MPa	2MPa	1.7 to 2.94 MPa	0.1575MPa	NA	0.1575MPa	NA	NA	2.15 or 2.65 MPa	2,94 MPa	0.9 MPa	2.68 MPa	2.94 MPa
**Mechanical index**	NA	NA	NA	1.3	1.41	1.7	0.16	NA	0.16	NA	NA	1.7 or 2.1	1.7	0.9	1.9	1.7
**Pulse duration**	2 msec	2 msec	4 msec	0.33msec	0.2msec	NA	2 msec	2 msec	2 msec	NA	NA	0.005 or 0.020 msec	NA	NA	NA	NA
**Pulse repetition frequency**	100 Hz	100 Hz	50 Hz	150 Hz	50Hz	NA	100 Hz	100 Hz	100 Hz	NA	NA	8 Hz	NA	NA	NA	NA
**Total treatment Duration (US)**	24 min	24 min	30 min	30 min	30 min	60 min	60 min	60 min	60 min	30 min	120 min	30 min	60 min	20 min	30 min	60 minutes

MI: Mechanical index; NA: Not available; Isata: spatial average temporal average intensity; Ispta: Spatial peak temporal average intensity.

### Thrombolytic drugs co-administered with the microbubbles

Table 4 summarizes the thrombolytic drugs co-administered with the MB.

### Evaluation criteria

#### Efficacy

TIMI (Thrombolysis in Myocardial Infarction), TIBI (Thrombolysis in Brain Infarction), custom patency, or de-clotting scores were used to assess the quality of arterial recanalization with angiography [18, 19, 24–26]. Gao et al. [20] used MRI to assess restoration of blood flow. Ren et al. [30] used US imaging and pulsed wave Doppler to assess recanalization. Other coagulation parameters used included activated partial thromboplastin time [26], activated clotting time [19], prothrombin time [26], fibrinogen [26, 27] and D-dimer [26]. Alonso et al. and Tomkins et al. [27, 31] examined thrombi in treated arteries using histology. Liu et al. [24] determined changes in S100B astroglial protein levels in blood were a marker of cerebral damage [34]. Diffusion-weighted sequences and phosphorus spectroscopy were used by Moumouh et al. [29] to assess and to quantify the extent of cerebral infarct. Nedelmann et al. [32] used MRI T2 relaxation time as indicator of edema formation. Cerebral infarct volume was assessed by eight studies as a treatment outcome [21–25, 28, 31, 32]. Nedelmann et al. and Schleicher et al. [32, 33] evaluated vascular volumes with microcomputed tomography. Finally, four studies [21, 22, 28, 32] used functional tests (as a marker of neurological improvement) after STL or rtPA-STL treatment.

#### Safety

Nine studies used histological analysis to assess intracranial hemorrhage (ICH) [20–25, 28, 32, 33].

### Treatment outcomes

Table 6 summarizes the treatment conditions and corresponding treatment outcomes of the 16 studies.

**Table 6:** Conditions undergoing testing and main outcomes (p < 0.05) of the 16 studies.

	References	Conditions tested	Positive outcomes (criteria)
**STL efficacy**	Wang et al. (2008)	- IA TSTL - IA saline control - IV TSTL - IV STL - US	- IA TSTL> IV TSTL (declotting time, success rate) - IA or IV TSTL > US (declotting time, and TIMI) - IA or IV TSTL > IA saline control - IV TSTL > IV STL (declotting time, TIMI)
	Moumouh et al. (2010)	- STL - RtPA	- STL > RtPA (Pcr/Pi (estimation of the oxidative phosphorylation metabolism))
	Alonso et al. (2009)	- IV STL with TMB - IV STL - Saline/US	- IV STL with TMB > IV STL (D-Dimer) - IV STL with TMB > US (D-Dimer)
	Culp et al. (2003)	- IA STL - IA Saline/US	- IA STL > IA Saline/US (declotting score, flow scores, success at 24 min)
	Culp et al. (2004)	- IV TSTL - IV eptifibatide/US - IV saline/US	- IV TSTL > IV eptifibatide/US (angiographic success, declotting score) - IV TSTL > IV saline/US (angiographic success, declotting score)
**Efficacy of rtPA-STL**	Tomkins et al. (2015)	- RtPA - RtPA-STL - Saline	No recanalization
	Ren et al. (2015)	(1) Control (2) RtPA (3) STL (4) RtPA-STL (5) ½ RtPA-STL	- After 10 min of treatment: RtPA-STL > contrl; RtPA; STL (Recanalization rate) - After 20 min of treatment: All conditions (except STL) induced a significant recanalization versus control (recanalization rate)
**Safety and efficacy of STL**	Culp et al. (2011)	- Control - RtPA - RtPA/US - Lipid STL - Albumin STL - TSTL	- All MB STL > control, rtPA (infarct volume, SB-100 rate), Pooled analysis with all type of MB (Lipid, Albumin, TMB)
	Liu et al. (2012)	- Urokinase - Urokinase-STL	- Urokinase-STL > Urokinase (infarct volume) - Urokinase-STL > Urokinase (mean flow velocity at 120 min)
	Ren et al. (2012)	- IA STL - IA TSTL - IV STL - IV TSTL - IA rtPA - IA saline/US	- IA TSTL > IA Saline/US (patency score, TIBI) - IA TSTL > IA STL (patency score, TIBI) - IA TSTL > IA Saline/US (recanalization rate) - IA rtPA > IA Saline/US (recanalization rate) - IA TSTL > IA STL (recanalization rate)
	Gao et al. (2014)	- (1) 1.7MHz, 1.7 MI, 20 μs pulse STL - (2) 1.6MHz, 2.4 MI, 5 μs pulse STL - (3) 1.6MHz, 2.4 MI, 5 μs pulse US	- (2)>(3) (after 24H, relatives changes in cerebral blood flow) - (2)>(1) (complete recanalization of ipsi and contralateral internal carotid and middle cerebral artery)
**Safety and efficacy of rtPA-STL**	Nedelmann et al. (2010)	Substudy 1: Efficacy - Euthanization directly after reperfusion (control 1) - Euthanization 60 min after reperfusion (control 2) - RtPA - RtPA-STL - STL - RtPA+US Substudy 2: Safety - RtPA - RtPA-STL Substudy 3: Temperature evaluation RtPA-STL	Substudy 1: Comparison between right and left hemisphere: the right hemisphere is submitted to a transient vascular occlusion of the middle cerebral artery. - Control 1 > healthy hemisphere (impaired vascular volume p<0.001) - Control 2 > healthy hemisphere (impaired vascular volume p<0.001) - RtPA > healthy hemisphere (impaired vascular volume, p<0.003) - RtPA-STL = Healthy hemisphere (impaired vascular volume, p<0.6) - US > healthy hemisphere (impaired vascular volume, p<0.001) - STL > healthy hemisphere (impaired vascular volume, p<0.04) Substudy 2: - RtPA-STL > RtPA (infarct volume, p<0.05) - RtPA-STL > RtPA (T2 relaxation, indicator of cerebral edema, p<0.05) Substudy 3: - No significant change of temperature from baseline
	Brown et al. (2011)	- control/US +/-MB - RtPA (0.1mg/kg) /US +/-MB - RtPA (0.3mg/kg) /US +/-MB - RtPA (0.8mg/kg) /US +/-MB - RtPA (0.9mg/kg) /US +/-MB	- RtPA*-STL > control/US/MB (infarct volume) - RtPA(0.8–0.9mg/kg)-STL> RtPA(0.8–0.9mg/kg) (lower ICH incidence) - RtPA*-STL > RtPA (all concentrations) (lower ICH incidence) - US/MB > control (infarct volume) - RtPA*-STL> control (infarct volume) - RtPA*-STL> rtPA* (reduced ICH incidence outside of strokes)
	Flores et al. (2011)	- Control - US - RtPA - RtPA/US - STL - RtPA-STL	- STL > RtPA/US; US; RtPA; RtPA-STL; control (decreased rate of ICH outside the infarct area) - RtPA/US; STL; RtPA; RtPA-STL > US (infarct volume)
	Lu et al. (2016)	- Control - STL - RtPA - RtPA-STL	- STL > control (red and white thrombi, cerebral infarct volume) - STL > RtPA (red thrombi, cerebral infarct volume) - RtPA-STL > RtPA (red thrombi, cerebral infarct volume) - STL > control (red and white thrombi, Neuroscore) - STL > RtPA (White thrombi, Neuroscore)
	Schleicher et al. (2016)	- Control - RtPA - RtPA-STL with 1/3 dose of MB - RtPA-STL with full dose of MB	Comparison between right (transient vascular occlusion) and left (healthy) hemisphere - Control > healthy hemisphere (impaired vascular volume, p<0.05) - RtPA > healthy hemisphere (impaired vascular volume, p<0.05) - RtPA-STL full MB + RtPA-STL 1/3MB = healthy hemisphere (impaired vascular volume, p>0.05) Comparison between condition - RtPA-STL full MB > Control (ischemic lesion volume, p = 0.044)

TSTL = STL using Targeted Microbubbles (TMB).

*Pooled analysis considering all concentrations of rtPA.

#### Efficacy

Three studies [18, 25, 26] used intra-arterial (IA) administration of MBs. Wang et al. [26] showed that STL using IA administration of targeted microbubbles (TMBs) produced superior de-clotting scores as compared to IV injection of TMBs. Similarly, Ren et al. [25] showed that TMBs induced better TIBI patency scores and efficient recanalization rates than IA administered non-targeted MBs. Culp et al. [19] obtained better de-clotting scores for STL using TMBs when compared to US + eptifibatide and US + saline. Using D-dimer dosage, Alonso et al. [27] demonstrated that rtPA-STL with TMBs caused higher efficient thrombi disintegration than STL or US alone. Culp et al. [22] reported lower infarct volume for STL with TMBs when compared to untreated controls. Nedelmann et al. and Shleicher et al. [32, 33] found no significant difference between the vascular volume of the hemisphere that experienced transient ischemia and rtPA-STL when compared to the healthy hemisphere.

With regard to brain damage, Brown et al. [21] noted a significant reduction in infarct brain volume for rtPA-STL when compared to STL or control animals. Similarly, Lu et al. [28] reported a reduction in infarct brain volume for STL and rtPA-STL on white microthrombi compared to rtPA alone and rtPA-STL on red microthrombi compared to rtPA alone. Moreover, Liu et al. [24] described a significant reduction in infarct brain volume for STL + urokinase when compared to urokinase treatment alone (p = 0.025). Nedelmann et al. [32] showed a lower infarct volume with a lower edema volume using rtPA-STL compared to rtPA treatment alone. In addition, Schleicher et al. [33] reported also a lower infarct volume using rtPA-STL compared to rtPA alone. Ren et al. [30] reported improved recanalization rates with rtPA-STL as compared to STL alone or rtPA alone after 10 minutes of treatment. However, Tomkins et al. [31] reported a complete absence of recanalization for rtPA-STL treatment on platelet-rich thrombi. In addition, functional tests performed in the studies by Brown et al., Culp et al., and Lu et al. [21, 22, 28] showed no noticeable differences between treatment regimens. Nevertheless, Lu et al. [28] reported better neurological scores for STL when compared to rtPA alone for the treatment of white microthrombi emboli.

#### Safety

Flores et al. [23] reported fewer hemorrhages outside the infarct zone for STL when compared to rtPA-STL, US + rtPA, rtPA alone, and US alone (*p* < 0.004). Brown et al. [21] found lower incidences of ICH not only for rtPA-STL compared to rtPA alone but also for the rtPA concentration of 0.8–0.9 mg/kg. The authors also reported reduced incidence of ICH outside of stroke zones when using rtPA-STL in comparison to rtPA (*p* = 0.005) alone, when the results obtained with all concentrations of rtPA were combined. All other studies showed no noticeable differences in the incidence of cerebral hemorrhage using rtPA-STL, STL alone, and rtPA alone.

### Quality assessment

The average CAMARADES criteria were 4.68 (± 1.08) for the 10 criteria tested, as summarized in Table 7.

**Table 7:** Quality assessment of the studies using the collaborative approach to meta-analysis and review of Animal Data from Experimental Studies (CAMARADES) checklist items.

CAMARADES CHECKLIST	Culp et al. (2003)	Culp et al. (2004)	Wang et al. (2008)	Alonso et al. (2009)	Moumouh et al. (2010)	Nedelmann et al. (2010)	Culp et al. (2011)	Brown et al. (2011)	Flores et al. (2011)	Ren et al. (2012)	Liu et al. (2012)	Gao et al. (2014)	Tomkins et al. (2015)	Ren et al. (2015)	Lu et al. (2016)	Schleicher et al. (2016)
**Publication in peer-reviewed journal**	Y	Y	Y	Y	Y	Y	Y	Y	Y	Y	Y	Y	Y	Y	Y	Y
**Statement of control of temperature**	N	N	N	Y	Y	Y	N	N	N	N	N	Y	Y	N	N	Y
**Randomization of treatment or control**	Y	Y	Y	N	N	N	Y	Y	Y	Y	Y	Y	N	N	N	N
**Allocation concealment**	Y	N	N	N	N	N	N	N	N	N	N	N	N	N	N	N
**Blinded assessment of outcome**	Y	N	Y	N	N	Y	N	Y	N	Y	N	N	N	N	N	Y
**Avoidance of anesthetics with marked intrinsic properties**	Y	Y	Y	Y	Y	Y	N	Y	N	Y	N	N	Y	N	Y	Y
**Use of animals with hypertension or diabetes**	N	N	N	N	N	N	N	N	N	N	N	N	Y	N	N	N
**Sample size calculation**	N	N	N	N	N	N	N	N	N	N	N	N	N	N	N	N
**Statement of compliance with regulatory requirements**	Y	Y	Y	Y	Y	Y	Y	Y	Y	Y	Y	Y	Y	N	Y	Y
**Statement regarding possible conflict of interest**	N	N	N	Y	N	Y	Y	N	N	Y	Y	N	Y	N	Y	Y
**Total (on 10)**	6	4	5	5	4	6	4	5	3	6	4	4	6	3	4	6

Owing to considerable heterogeneity in the reported methods and data, meta-analysis was not feasible. As a result, no statistical examination was performed.

## Discussion

### Efficacy

This systematic review highlights a number of positive outcomes in terms of efficacy. Numerous criteria are evaluated throughout the studies. Half of the studies (8 of 16) assess cerebral infarct volume, while four studies use functional recovery tests and neurological scores. The most commonly used criteria are the recanalization rate and the quality of the vascular flow stream. However, discordances have been reported between apparent positive recanalization and favorable outcomes. Ren et al. [25] highlight the limit of this pure blood flow restoration assessment by showing a significant difference in recanalization scores and quality of the flow stream, but not in the infarcted brain volume. Infarcted brain volume and neurological score are the two main outcomes used in clinical trials to assess thrombolytic therapy [2–4]. Similar results are described in humans in the CLOTBUST (Combined Lysis Of Thrombus in Brain ischemia using transcranial Ultrasound and Systemic TPA) study [35], which has raised the need to consider clinical outcome as the pivotal end-point for all studies. This observation is in line with the Stroke Therapy Academic Industry Roundtable (STAIR) group recommendations [36].

Different stroke models are used to investigate the efficacy of STL—13 studies assess it on a thromboembolic model with a blood clot, and 3 studies assess it on microvasculature impairment (transient occlusion or microthrombi emboli). It appears that a clear benefit exists with the use of rtPA-STL in microvasculature impairment (transient occlusion and red or white micro-thrombi) compared to control or rtPA alone. White thrombi seem to be more resistant to STL or rtPA-STL [31]. Studies involving a thromboembolic model, present more heterogeneous results, as only 3 studies demonstrated a benefit of STL or rtPA-STL versus rtPA alone on clinical criteria. These discrepancies can be partly due to the variation in treatment initiation times highlighted in this review. Indeed, ischemia induce an inflammatory response in the salvageable penumbra area which is responsible for the secondary necrosis of this area [37]. Furthermore, this response could also lead to a reperfusion injury syndrome inducing oedema and hemorrhages [38]. These phenomenon are presumably absent immediately after vascular occlusion [39].

In terms of MBs used and US parameters, TMBs (Targeted Microbubbles) are more efficient than MBs, but none of the studies have tested the association of rtPA-TMBs (rtPA associated with TMB) versus rtPA. With the data available, one cannot conclude on the clinical interest of TMBs. Further investigations are required to evaluate the therapeutic efficacy of TMBs for the STL. The heterogeneity of US parameters used does not allow us to conclude which combination is the more efficient or if a specific combination of US parameters is giving better benefit to risk ratio in the specific pathophysiology of stroke. The only parameter between studies that seems to be consensual is the use of an US frequency predominantly in the Megahertz (MHz) range, probably to avoid standing waves [40] which has been supposed to be responsible of a high rate of ICH through an augmentation of peak negative pressure in the Transcranial Low-Frequency Ultrasound-Mediated Thrombolysis in Brain Ischemia (TRUMBI) study with the use of a US frequency of 300 kiloHertz (kHz) wi wi thout MBs in humans [41].

### Safety

Existing clinical STL data has shown that cerebral hemorrhage is the major side effect in terms of safety. Two studies report that rtPA-STL leads to fewer cerebral hemorrhages outside the infarcted zone. These two studies show no significant efficacy of rtPA-STL versus rtPA alone [21, 23]. Based on these limited results, it may be inappropriate to draw conclusions on the safety of STL or rtPA-STL versus rtPA alone; and more generally on the benefit to risk ratio of this therapeutic approach. Compared to clinical trials, the results reported in this review are surprising, as none of the studies revealed a higher rate of cerebral hemorrhage versus control, even with the use of rtPA alone, which is known to significantly increase the rates of cerebral hemorrhage in clinical studies [2]. Some hypotheses can be made to explain the following: publication bias (e.g., the absence of the publication of studies showing a higher rate of cerebral hemorrhage with STL), the use of animal models with no risk factor of bleeding (e.g., aging or hypertension), and the absence of ischemia before treatment.

### Clinical translation/perspectives

The studies reviewed in this report use a thromboembolic stroke model or a microvasculature impairment model (by transient ischemia or deployment of microthrombi). Stroke in humans is due to different pathophysiological mechanisms associated with cardiovascular (CV) risk factors that are summarized in the TOAST (Trial of ORG 10172 in Acute Stroke Treatment) classification [42]. The use of an animal model closer phylogenetically to humans, such as gyrencephalic species, associated with CV risk factors (e.g., aging and hypertension) could bring more consistent data, as suggested by the recommendations for animal testing in stroke [36]. Some specificity of the human pathophysiology (e.g., the human skull) may be considered to ensure a clinical translation. Indeed, the interactions between the human skull and US beam are responsible for acoustic processes including standing waves, degradation of US focalization, and degradation of beam shape. High frequency ultrasound will also lead to more attenuation and heating [43]. These processes depend on the frequency used, the position of the US transducer, and the length of the US wave [44]. The TRUMBI trial [41] reports higher rates of cerebral hemorrhage, which are probably caused by standing waves. Standing waves field happens mainly with the use of low frequency US, and lower the cavitation threshold in comparison with a progressive wave field [45]. In STL, MBs act as nuclei in the medium and can induce different effects depending on the acoustic conditions. While MB can be used to induce stable cavitation, which enhance rtPA thrombolysis without disrupting the blood brain barrier [46, 47], MB also lower the threshold of inertial cavitation which is able to induce a putative cerebral hemorrhage. Blood brain barrier disruption is induced by STL in certain acoustic conditions, which can also explain ICH (specifically in presence of rtPA, which is known to be neurotoxic and to cause hemorrhage) [44, 48, 49]. To avoid these adverse effects, some solutions have been developed to create a controlled intracranial US field that have not yet been tested in STL for IS. For example, it is now possible to monitor inertial cavitation in the brain in real time by using a passive cavitation detector [50]. Wright et al. were able to monitor cavitation during sonothrombolysis with high-intensity focused ultrasound in a rabbit femoral artery [51]. Frequency modulation is a solution to lower the occurrence of standing waves [52] through the use of higher US frequencies and short emission duration [44].

Associating STL with thrombolytic drugs or with mechanical thrombi retrieval is not well defined and needs to be fully explored [4]. Similarly, the question of whether STL without thrombolytic drugs or STL with reduced MBs dose leads to better safety ratios in certain clinical situations (e.g., when rtPA is contraindicated) needs to be addressed. Furthermore, the CLOTBUST clinical trial (using US alone) has pointed out that insonation remains operator dependent. A couple of solutions exist—the first one is the MRI-guidance (MRI = Magnetic resonance Imaging) of US [53, 54] and, the second one is the use of an operator independent US device (as reported in the CLOTBUST-HF trial) [55]. At this time, none of these solutions have been evaluated with STL in IS.

The use of TMBs seems promising, but TMBs are not yet validated for clinical use even for diagnostic purpose. In addition, regulatory complications that have to be overcome to get clinical approval of a new type of MBs agent will delay the clinical translation of this therapeutic approach. The concentration of MBs to be used is not consensual and appears as a critical safety element. Indeed, Transcranial ultrasound in clinical sonothrombolysis (TUCSON) trial [7] indicate that an association between the concentration of MBs and the rate of cerebral hemorrhage could exist (for the same US parameters). Recently McMahon et al. demonstrated that acute inflammatory response (which lead to microhemorrhage) following blood-brain-barrier opening with focused ultrasound and microbubbles is dependent on microbubble dose [56].

### Limitations

The present review represents the first systematic evaluation of in vivo preclinical studies with respect to STL treatment of experimental IS. Although our systematic screening of the relevant databases identified > 1000 articles on STL, only 16 studies met the inclusion criteria. We decide to exclude studies involving coronary or peripheral arteries, as brain has a specific vulnerability to ischemia and hemorrhage that is not present in cardiac muscle or other organs.

We regroup the microthrombi spreading and transient vascular occlusion under the term “microvasculature impairment model”, because authors stated that these models refer to the human “no reflow phenomenon” (where the distal arterioles stay occluded after a large vessel recanalization).

Meta-analysis is not feasible because of the heterogeneity of the studies reviewed.

Our quality evaluation reveals biases, which can be easily corrected in future investigations. 3 studies were published before or the year after the publication of the CAMARADES criteria. The absence of power calculations of the sample sizes in all studies reviewed means that some of the negative results reported may be linked to a default in statistical power. Information such as temperature of the animal is not always monitored, while hypothermia can lead to neuroprotection. Similarly, free neuroprotective anaesthetics can be implemented because their effects can falsely influence treatment outcomes.

## Conclusions

STL is a complex treatment requiring a multidisciplinary scientific approach. Nevertheless, selected preclinical studies have shown that STL has a substantial thrombolytic effect. Data available on safety are limited and more preclinical evaluation is needed to demonstrate a clear benefit to risk ratio on clinical endpoints, as compared to approved treatment. We suggest testing frequencies in the range from sub-MHz to 1 MHz, with intensities below the threshold of inertial cavitation, so to favor stable cavitation of microbubbles. The use of frequency modulation and short pulse durations should lessen the probability of the generation of standing wave. While TMB demonstrated a better efficacy than microbubbles, the use of clinically-approved microbubble may accelerate clinical translation. Further investigations on the doses of microbubbles and their association with thrombolytic drugs (mainly rtPA) are needed to improve the therapeutic benefit of STL. Although this therapeutic option could be used for large artery occlusion, this approach should also be beneficial in the situation of microcirculation impairment (e.g., after thrombectomy). Moreover, the future explorations on the safety of STL are essential. To achieve this objective, animal models, which reproduce the human pathophysiology (i.e., older animals, cardio-vascular risk factors, significant duration of ischemia) should be used. Finally, MRI guidance and cavitation detection have not been evaluated in ischemic stroke yet but their use in STL look promising to guarantee a good efficacy/safety profile.

## Supporting information

S1 PRISMA Checklist PRISMA checklist of the systematic review following the preferred reporting items for systematic reviews and meta-analyses. [17].
(DOC)

S1 File S1 File contains the full electronic search strategy for the two electronical databases. (DOCX)
