# Supplementary material for: Microbubbles combined with ultrasound therapy in ischemic stroke: A systematic review of in-vivo preclinical studies
Source: PLoS One. 2018 Feb 8;13(2):e0191788. doi: 10.1371/journal.pone.0191788 (PMC5805249; doi:10.1371/journal.pone.0191788)
Supplement: S1 File — (DOCX) [file pone.0191788.s002.docx]

**Research equation :**

**Pubmed®:**

(((((((stroke[MeSH Terms] OR fibrinolytic drugs[Mesh Terms] OR thrombolytic OR Thrombolytic agents[MeSH Terms] OR thrombolytic drugs[MeSH Terms]) AND (Microbubbles[MeSH Terms] OR Microspheres[MeSH Terms]) AND ("French"[language] OR "English"[language])) OR Sonothrombolysis))) AND ("1995"[Date - Publication] : "3000"[Date - Publication])))

**Web of science®:**

TOPIC: (thrombolytic therapy OR Stroke) AND TOPIC: (microbubbles OR Microspheres) OR TOPIC: (sonothrombolysis) OR TOPIC: (ultrasound contrast agents AND stroke) OR TOPIC: (ultrasound and thrombolysis)
